# Response assessment using ^68^Ga-PSMA ligand PET in patients undergoing ^177^Lu-PSMA radioligand therapy for metastatic castration-resistant prostate cancer

**DOI:** 10.1007/s00259-018-4236-4

**Published:** 2018-12-19

**Authors:** Bernhard Grubmüller, Daniela Senn, Gero Kramer, Pascal Baltzer, David D’Andrea, Karl Hermann Grubmüller, Markus Mitterhauser, Harald Eidherr, Alexander R. Haug, Wolfgang Wadsak, Sarah Pfaff, Shahrokh F. Shariat, Marcus Hacker, Markus Hartenbach

**Affiliations:** 1 Department of Urology, Medical University of Vienna, Vienna, Austria; 2 Working Group of Diagnostic Imaging in Urology, Austrian Society of Urology, Vienna, Austria; 3 Department of Biomedical Imaging and Image Guided Therapy, Division of Nuclear Medicine, Medical University of Vienna, Währinger Gürtel 18-20, 1090 Vienna, Austria; 4 Department of Biomedical Imaging and Image guided Therapy, Division of General and Pediatric Radiology, Medical University of Vienna, Vienna, Austria; 5 Department of Urology and Andrology, University Hospital Krems, Karl Landsteiner University of Health Sciences, Krems, Austria; 6 Ludwig Boltzmann Institute for Applied Diagnostics, Vienna, Austria; 7 Center for Biomarker Research in Medicine, CBmed GmbH, Graz, Austria; 8 Department of Urology, University of Texas Southwestern, Dallas, TX USA; 9 Department of Urology and Division of Medical Oncology, Weill Medical College of Cornell University, New York, NY USA

**Keywords:** Hybrid imaging, PET/MRI, PET/CT, Metastatic prostate cancer, PSMA ligand

## Abstract

**Purpose:**

The first aim of this study was to evaluate ^68^Ga-PSMA^HBED-CC^ conjugate 11 positron emission tomography (PSMA PET) parameters for assessment of response to ^177^Lu-PSMA-617 radioligand therapy (RLT) in patients with metastatic castration-resistant prostate cancer (mCRPC). The second aim was to investigate factors associated with overall survival (OS).

**Methods:**

We retrospectively assessed mean standardized uptake values (SUVmean) and total tumor volumes (TTV) on PSMA PET in 38 of 55 mCRPC patients before and after RLT. PSA testing and PSMA PET/CT(MRI) imaging were performed during the 8 weeks before and the 6 weeks after RLT. PSMA PET and CT(MRI) images were reviewed separately according to the modified PET Response Criteria in Solid Tumors (mPERCIST) and RECIST1.1. The results were compared with PSA responses. Associations between OS and the RECIST evaluation and changes in SUVmean, TTV, and PSA, CRP, LDH, hemoglobin and ALP levels were determined in a univariable survival analysis.

**Results:**

The median PSA level at the time of pretherapy PSMA PET/CT(MRI) was 60.8 ng/ml (IQR 15.4, 264.2 ng/ml). After RLT the median PSA level decreased by 44%, TTV by 45.1%, SUVmean by 25.8% and RECIST by 11.3%. A PSA response was seen in 18 patients (47.4%), stable disease in 12 (31.6%) and progressive disease in 8 (21.1%). Contrary to the changes in SUVmean and the RECIST evaluation, the change in TTV was significantly associated with PSA response (*p* = 0.15, *p* = 0.58, and *p* < 0.001, respectively). After a median follow-up of 17 months (IQR 8.0, 24.2 months), 11 patients (28.9%) had died of their prostate cancer. The changes in both TTV and PSA levels were associated with OS (HR 1.001, 95% CI 1–1.003, *p* = 0.04, and HR 1.004, 95% CI 1.001–1.008, *p* = 0.01, respectively), while the changes in SUVmean and the RECIST evaluation were not. The pre-therapy CRP level was also associated with OS (HR 1.07, 95% CI 1.009–1.14, *p* = 0.02).

**Conclusion:**

TTV on PSMA PET seems to be a reliable parameter for response assessment in mCRPC patients undergoing RLT and might overcome the limitations of RECIST in prostate cancer. Furthermore, the change in TTV was significantly associated with OS in our cohort.

## Introduction

Over recent decades life expectancy of patients with metastatic castration-resistant prostate cancer (mCRPC) has been prolonged by the emergence of novel therapies [1–6]. Prediction and monitoring of response to therapy allows selection of the ideal patients, and changes in therapy early in the resistance phase when necessary. Currently, the most commonly used tools for response assessment include cross-sectional abdominopelvic imaging with computed tomography (CT) or magnetic resonance imaging (MRI) according to the Response Evaluation Criteria in Solid Tumors (RECIST) [7], and bone scintigraphy (BS) together with serial assessments of changes in serum levels of prostate-specific antigen (PSA). Unfortunately, the current RECIST and even sometimes PSA levels show limited diagnostic and predictive accuracy in this disease state [8, 9]. Therefore, further parameters are needed in this context. Positron emission tomography (PET) has been shown to be a beneficial addition to morphological imaging, as it adds information about the molecular processes in tissues [10]. Indeed, PET/CT, as a hybrid imaging modality, shows superior diagnostic performance compared with standard imaging allowing accurate response assessment in other tumor types [11, 12].

In prostate cancer, ^68^Ga-PSMA^HBED-CC^ conjugate 11 (PSMA 11) PET has started to revolutionize imaging, improving primary staging and the detection of biochemical recurrence [13, 14]. The use of PSMA 11 PET for response assessment is in accordance with the theranostic concept, as both radiopharmaceuticals, PSMA 11 and the therapeutic agent ^177^Lu-PSMA 617 (PSMA 617), target transmembrane folate hydrolase [15] and therefore imaging before and after therapy provides direct information about therapeutic effects. However, to date, minimal data are available for PSMA 11 PET and its potential benefits for response assessment in patients with mCRPC. A recently published study in 23 patients treated with docetaxel showed that PSMA 11 PET/CT provides more accurate response assessment than conventional imaging [16]. We hypothesized that PSMA 11 PET would also provide better response assessment than conventional imaging in mCRPC patients treated with PSMA 617 radioligand therapy (RLT). We therefore investigated the potential of PSMA 11 PET parameters for the assessment of response to RLT in patients with mCRPC. Our second aim was to determine the parameters that were best associated with overall survival (OS) in this mCRPC cohort. We compared the predictive accuracy of PSMA 11 PET with that of standard imaging and laboratory values.

## Materials and methods

### Patients and therapy

This was a retrospective study in mCRPC patients undergoing PSMA 11 PET/MRI or PET/CT before and after RLT between January 2015 and January 2018. Patients eligible for this evaluation had to have a full PSMA 11 PET/CT(MRI) dataset before and after RLT and to have the respective laboratory values available. All patients received three administrations of 7.4 GBq PSMA 617 every 4 weeks according to a standardized protocol at our clinic. Originally, all patients were scheduled to undergo PSMA 11 PET/MRI. However, patients with implants contraindicated for a MRI and those with claustrophobia or pain underwent PSMA 11 PET/CT. PSMA 11 PET imaging and PSA determination were performed during the 8 weeks before starting RLT (PET1/RECIST1) and during the 6 weeks after the third RLT cycle (PET2/RECIST2). In addition to the standard baseline characteristics of the patients, the following pretreatment laboratory values were chosen for the survival analysis: PSA [17], hemoglobin [17], alkaline phosphatase (ALP) [17], C-reactive protein (CRP) [18] and lactate dehydrogenase (LDH) [19]. ^177^Lu-PSMA 617 was used according to section 7(6a) of the Austrian pharmaceutical law. This study retrospectively evaluated ^68^Ga-PSMA 11 PET assessment of response to therapy administered on a compassionate use basis while performing a prospective clinical trial of this radiopharmaceutical at the same institution (clinicaltrials.gov NCT02659527). All reported investigations were conducted in accordance with the principles of the Declaration of Helsinki and national regulations. The study was approved by the local Ethics Committee (permit 1158/2018).

### Imaging protocol, analyses and evaluation of PET parameters

#### PSMA 11 PET/MRI protocol

Patients received an intravenous injection of 2 MBq/kg body weight ^68^Ga-PSMA^HBED-CC^ conjugate 11 60 min before the start of the PSMA 11 PET/MRI acquisition. PET/MRI was performed on a Biograph mMR system (Siemens, Germany), that consisted of a MRI-compatible PET detector integrated with a 3.0-T whole-body MRI scanner. The PET component used a three-dimensional (3D) acquisition technique and offered an axial field of view (FoV) of approximately 26 cm and a transverse FoV of 59 cm with a sensitivity of 13.2 cps/kBq. A partial body PET scan (skull base to thighs) was performed with four bed positions, 5 min sinogram mode each. PET images were reconstructed using three iterations and 21 subsets. MRI-based attenuation correction was applied using Dixon-VIBE sequences that comprised in-phase and opposed-phase as well as fat-saturated and water-saturated images.

Integrated 3-T MRI was performed with a diffusion-weighted imaging (DWI) sequence and a T1 VIBE sequence for partial body imaging simultaneously with PET, and a T1 TSE sequence, a T2 TIRM sequence and a T2 HASTE sequence for sagittal imaging of the spine after PET, with the following parameters. DWI sequence: *b*-values 50 and 800, TR 6,800 ms, TE 63 ms, six averages and one echo, flip angle 180°, matrix size 168 × 104, FoV 440 × 340 mm, slice thickness 6 mm with a 1.2-mm gap. T1 VIBE sequence: matrix size 195 × 320, in-plane resolution 1.6 × 1.2 × 3 mm, FoV 309 × 380 mm, TR 4.56 ms, TE 2.03 ms. T1 TSE sequence: matrix size 320 × 320, in-plane resolution 1.4 × 1.1 × 3 mm, FoV 263 × 350 mm, TR 666 ms, TE 9.6 ms. T2 TIRM sequence: matrix size 320 × 320, in-plane resolution 1.4 × 1.1 × 3 mm, FoV 263 × 350 mm, TR 3,500 ms, TE 43 ms. T2 HASTE sequence: matrix size 256 × 256, in-plane resolution 1.56 × 1.5 × 6 mm, FoV 380 × 380 mm, TR 1,400 ms, TE 121 ms.

#### PSMA 11 PET/CT protocol

PSMA 11 PET/CT was performed from the vertex to the upper thighs using a 64-row, multidetector hybrid system (Biograph TruePoint 64; Siemens, Erlangen, Germany), with an axial FoV of 216 mm, a PET sensitivity of 7.6 cps/kBq, and a transaxial PET resolution of 4–5 mm (full-width at half-maximum). PET was performed 60 min after intravenous administration of 2 MBq/kg body weight ^68^Ga-PSMA^HBED-CC^ conjugate 11 with 4 min per bed position, four iterations/21 subsets, slice thickness 5 mm and matrix size 168 × 168, using the point-spread function-based reconstruction algorithm TrueX. CT maps were used for PET attenuation correction. Venous-phase contrast-enhanced CT was performed after intravenous injection of 100 ml of a tri-iodinated, non-ionic contrast medium at a rate of 2 ml/s, with tube voltage 120 kV, tube current 230 mAs, collimation 24 × 1.2 mm, slice thickness 3 mm at a 2-mm increment, and matrix size 512 × 512.

#### Image evaluation

**Fig. 1:**
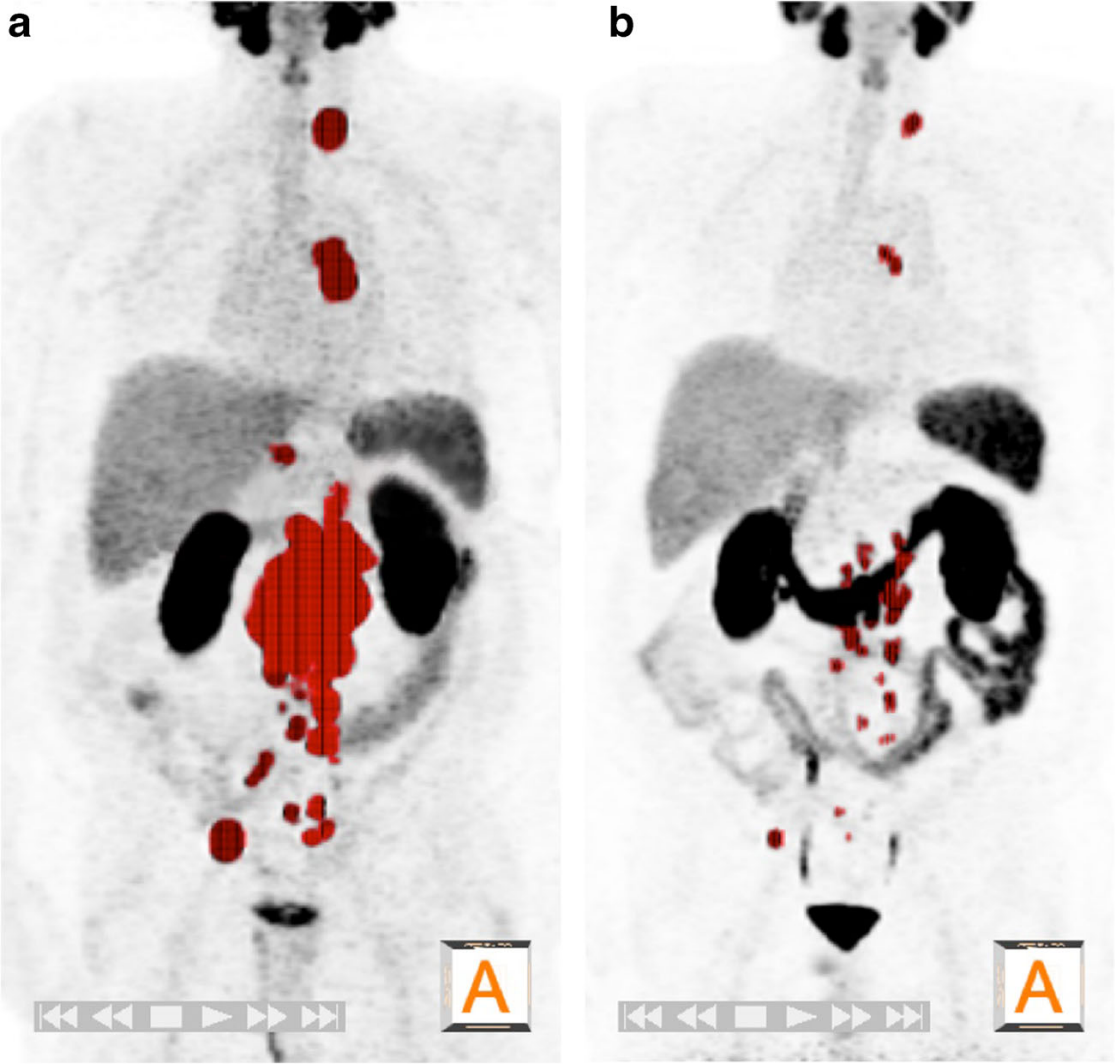
A 74-year-old patient with castration-resistant metastatic prostate cancer (mCRPC) after treatment with enzalutamide and docetaxel. **a**^68^Ga-PSMA 11 PET image before the first ^177^Lu-PSMA-617 radioligand therapy (RLT) in December 2015 demonstrates extended pelvic, abdominal and thoracic lymph node metastasis. The total tumor volume (TTV) measured semiautomatically as described in Materials and methods is marked *red* in the maximum intensity projection (MIP) 3D image. The PSA level at the time of imaging was 597 ng/ml, and the TTV was 359 ml. **b** After three cycles of RLT (7.4 GBq each), the PSA level had significantly decreased (23.1 ng/ml) and the TTV had reduced (43 ml), demonstrating a partial response. The patient received a further three cycles of RLT that resulted in a biochemical complete response (PSA 0.01 ng/ml, January 2018)

PSMA 11 PET parameters were evaluated using Hermes Hybrid3D (Hermes Medical Solutions, Stockholm). The total tumor volume (TTV) was derived using a threshold-based volume of interest (VOI) extracted from the complete FoV. The lower threshold was defined as the mean SUV derived from a cubic 10 × 10 × 10 voxel reference VOI of the liver plus 20% to avoid most of the nonspecific and physiological PSMA 11 uptake [20]. The reference VOI was manually drawn by a single investigator avoiding the inclusion of major intrahepatic vessels based on CT/MRI. A program-inherent segmentation algorithm was then applied to the threshold-derived VOI, enabling the deletion of the most common significant noncancer uptake areas (kidney, salivary glands, gut, spleen, bladder). Nonspecific uptake still present was then cropped manually (Fig. 1). The evaluated SUVmean was then taken from the derived TTV/VOI.

CT/MRI alone was separately assessed according to RECIST 1.1 [7] by a uroradiologist (P.B.) using AGFA IMPAXX EE software.

### Assessment of therapy response

#### Biochemical

The biochemical response (BR) measured in terms of serum PSA levels served as the standard of reference for response assessment [16]. Biochemical complete response (CR) was defined as a PSA level of 0 ng/ml after RLT, partial response (PR) as a decrease in serum PSA level of ≥50% from the baseline level, progressive disease (PD) as an increase in serum PSA level of ≥25% from the baseline level. Changes in PSA level after therapy of between −50% and +25% were considered to indicate stable disease (SD) [11, 16].

#### Radiographic

Radiographic response (RR) was assessed separately for PSMA 11 PET and MRI (or CT). The PET2 evaluation was compared with the PET1 evaluation and is reported as RR(PET). As described previously by Seitz et al. [16], changes in PET parameters after RLT were interpreted according to the modified PET Response Criteria in Solid Tumors (PERCIST) 1.0 [21]. A lack of all PSMA 11-avid lesions on PET2 that were seen on PET1 was defined as CR, a decrease of summed SUVmean or TTV of ≥30% as PR, and new PSMA 11-avid lesions on PET2 or an increase in SUVmean or TTV of ≥30% as PD. Intermediate changes in SUVmean and TTV on PET2 of between −30% and +30% were considered to indicate SD.

MRI (CT) datasets were analysed according to RECIST 1.1 [7]. The findings of the RECIST evaluation are reported as RR(RECIST). The disappearance of all lesions was defined as CR, a decrease in the summed diameter of the index lesions of ≥30% as PR, and the appearance of new lesions or an increase in the summed diameter of the index lesions of ≥20% as PD. Changes in summed diameter of between −30% and +20% were considered to indicate SD.

### Follow-up

Due to the retrospective nature of the study, patient follow-up was not standardized. In general, patients underwent physical examination and laboratory testing monthly. Follow-up time was calculated from the date of the first RLT cycle. Patients who died were considered at the date of their last follow-up. The cause of death was as asserted by the treating physician.

### Statistical analyses

The univariable Cox proportional hazards model was used to test the associations between OS and changes in PSA level, TTV and SUVmean after therapy and the findings of the RECIST evaluation, and between OS and pretreatment laboratory values. Cohen’s kappa (*κ*) was used to measure interrater agreement between PSA and TTV response, SUVmean response and the findings of the RECIST evaluation. Kaplan-Meier estimates were used to evaluate differences in OS between patients with PSA/TTV CR and those with PR and between patients with PSA/TTV SD and those with PD. All tests were two-sided. The significance level was set to <0.05. Analyses were performed with R 3.4 (he R Foundation for Statistical Computing, Vienna, Austria).

## Results

### Patient characteristics

Of 55 patients who underwent RLT up to January 2018 (all of the patients underwent three cycles RLT), 38 had all necessary data available and were included in the analyses. All patients had androgen-deprivation therapy (ADT) plus abiraterone/enzalutamide and/or chemotherapy (docetaxel/cabazitaxel) before undergoing RLT. Baseline characteristics are shown in Table 1. The median serum PSA level at the time of PET1 was 60.8 ng/ml (IQR 15.4, 264.2 ng/ml). Eight patients (21.1%) had lymph node metastases only, another eight (21.1%) had bone metastases only, and 22 (57,9%) had lymph node and bone metastases, of whom six (15.8%) showed additional visceral metastases.

**Table 1:** Baseline characteristics of the 38 included patients staged using ^68^Ga-PSMA^HBED-CC^ conjugate 11 ligand PET/MRI(CT) before ^177^Lu-PSMA-617 radioligand therapy

Characteristic	Value
Age (years), median (IQR)	71.47 (67.30, 77.34)
Prior radical prostatectomy, *n* (%)	28 (73.7)
Prior primary radiotherapy, *n* (%)	5 (13.2)
Prior systemic therapy lines, *n* (%)	
≤ 2	11 (29)
≥ 3	27 (71)
ADT at imaging, *n* (%)	25 (65.8)
PET/CT, *n* (%)	6 (15.8)
PET/MRI, *n* (%)	32 (84.2)
Amount of used PSMA (MBq), median (IQR)	171.50 (160.75, 187.25)
Metastatic sites, *n* (%)	
Local recurrence	11 (28.9)
Lymph nodes	30 (78.9)
Bone	30 (78.9)
Viscera	6 (15.8)
Prostate-specific antigen (ng/ml), median (IQR)	60.8 (15.4, 264.2).
Hemoglobin (mg/dl), median (IQR)	11.8 (10.6, 13.2)
C-reactive protein (g/dl), median (IQR)	0.4 (0.2, 1.2)
Lactate dehydrogenase (U/L), median (IQR)	189.0 (172.0, 245.3)
Alkaline phosphatase (U/L), median (IQR)	68.0 (56.5, 123.3)

*IQR* interquartile range, *ADT* androgen-deprivation therapy, *PSA* prostate-specific antigen

### Response assessment of PET parameters compared to standard imaging

**Fig. 2:**
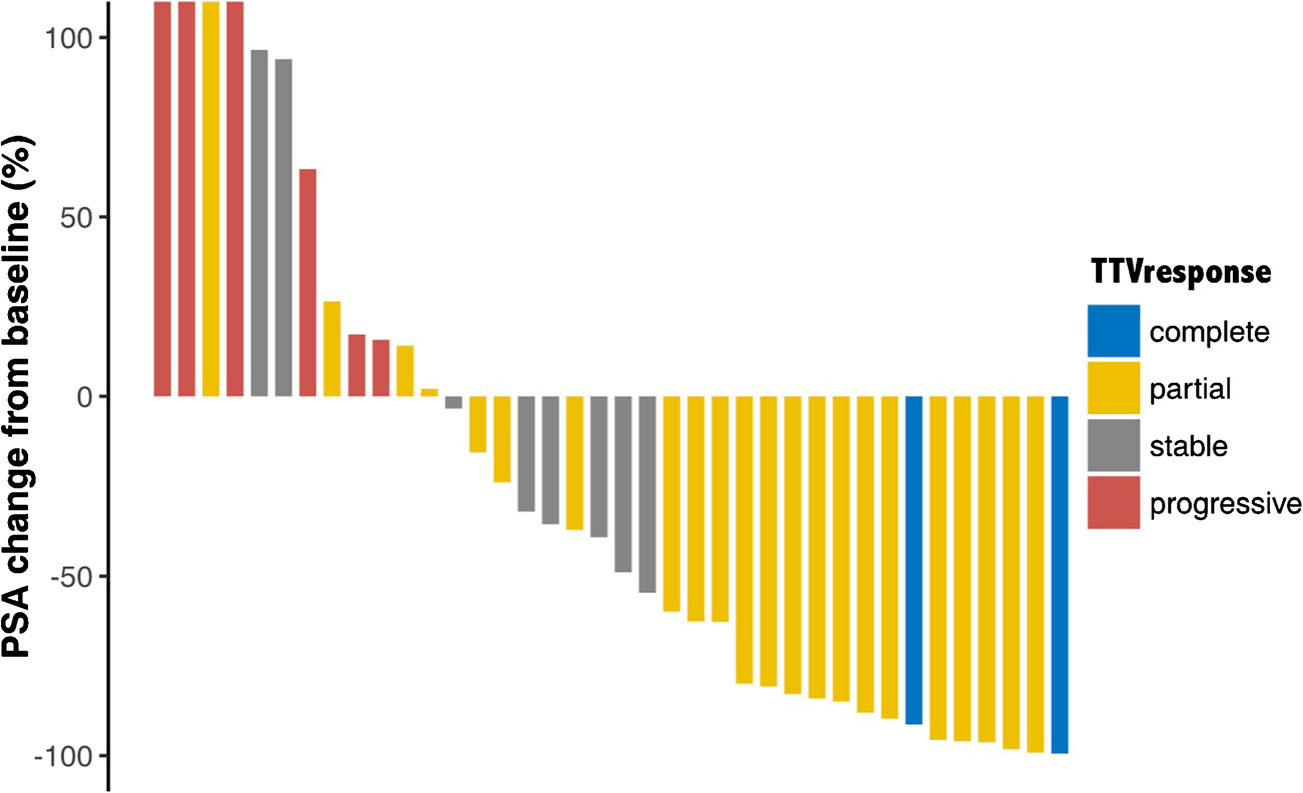
Percentage changes in PSA level from baseline in 38 patients undergoing ^177^Lu-PSMA-617 radioligand therapy in relation to the responses measured in terms of PSMA total tumor volume (TTVresponse)

Table 2 shows BR and RR(PET+RECIST) in relation to changes in PSA level, TTV and SUVmean and the RECIST evaluation. Concordance between BR and RR(PET) and RR(RECIST) in patients undergoing RLT is shown in Tables 3 and 4. Contrary to the change in SUVmean and the RECIST evaluation, change in TTV showed a significant correlation with the PSA response rate after therapy (*p* = 0.154, *p* = 0.583, *p* < 0.001, respectively; Fig. 2). Overall, 18 patients had a biochemical PR, which was concordant in 15 patients (83.3%) on PET2 measured as change in TTV. In two (11.1%) of the three patients with discrepant results the RR(PET) was CR on PET2, and both of these patients showed not just an excellent PSA response (PSA levels after therapy 0.61 ng/ml and 0.41 ng/ml, respectively), but also a CR as measured using RECIST and change in SUVmean. In the remaining patient (5.6%), RR(PET+RECIST) was SD. A further three RLT cycles were performed due to the PSA response and radiographic SD. However, this patient died within 15 months of the start of the initial RLT cycle.

**Table 2:** Biochemical and radiographic responses expressed as changes in PSA level, TTV, SUV and RECIST in 38 patients after ^177^Lu-PSMA-617 radioligand therapy

Response measure	Change after RLT (%), median (IQR)	Response, *n* (%)			
		Complete	Partial	Stable disease	Progressive disease
PSA	−43.98 (−84.67, 15.37)	0 (0.0)	18 (47.4)	12 (31.6)	8 (21.1)
TTV	−45.13 (−83.83, 4.41)	2 (5.3)	22 (57.9)	8 (21.1)	6 (15.8)
SUVmean^a^	−25.76 (−46.54, −6.14)	2 (5.3)	17 (44.7)	17 (44.7)	2 (5.3)
RECIST^b^	−11.25 (−33.94, 0.00)	1 (2.6)	9 (23.7)	15 (39.5)	5 (13.2)

*PSA* prostate-specific antigen, *TTV* PSMA total tumor volume, *SUVmean* mean PSMA standardized uptake value, *RECIST* Response Evaluation Criteria in Solid Tumors, *IQR* interquartile range, *RLT* radioligand therapy

^a^Liver SUVmean: 3.1 (IQR 2.5–4.1) before RLT, 3.2 (IQR 2.6–3.8) after three cycles of RLT

^b^Response evaluation was not possible in eight patients (21.1%) because they had no measurable target lesions according to RECIST 1.1

**Table 3:** Concordance between biochemical response and radiographic response (PET) in 38 patients undergoing ^177^Lu-PSMA-617 radioligand therapy

		TTV				SUVmean			
		Complete response	Partial response	Stable disease	Progressive disease	Complete response	Partial response	Stable disease	Progressive disease
PSA	Complete response	0	0	0	0	0	0	0	0
	Partial response	2	15	1	0	2	5	10	1
	Stable disease	0	5	5	2	0	8	4	0
	Progressive disease	0	2	2	4	0	4	3	1
		*κ* = 0.412, *p* < 0.001				*κ* = −0.159, *p* = 0.154			

*PSA* prostate-specific antigen, *TTV* PSMA total tumor volume, *SUVmean* mean PSMA standardized uptake value

**Table 4:** Concordance between biochemical response and radiographic response (RECIST) in 30 patients undergoing ^177^Lu-PSMA-617 radioligand therapy

		RECIST			
		Complete response	Partial response	Stable disease	Progressive disease
PSA	Complete response	0	0	0	0
	Partial response	1	6	8	0
	Stable disease	0	2	5	4
	Progressive disease	0	1	2	1
		*κ* = 0.069, *p* = 0.583			

*PSA* prostate-specific antigen, *RECIST* Response Evaluation Criteria in Solid Tumors

Biochemical SD after RLT was seen in 12 patients. Concordance between PET1 and PET2 was found in 5 of the 12 patients (41.6%) measured as change in TTV. Another 5 patients (41.6%) showed a PR on PET2. All of these patients also showed a decrease in PSA level, but in none of these patients did the decrease reach −50% for a biochemical PR per definition (decrease in PSA level of 2–37%). Two of the 12 patients (16.7%), one of whom died during follow-up, had PD on PET2. Conversely, these patients showed an increase in PSA level, but in none of these patients did the increase reach 25% for biochemical PD per definition (increase in PSA level of 15% and 17%, respectively). The response rates on PET2 were correct in four of eight patients (50%) with biochemical PD. Two patients (25%), of whom one died during follow-up, showed a radiographic SD on PET2 with an increase in TTV that did not reach the cut-off for PD. The remaining two patients (25%) had a radiographic PR as measured by TTV; both died during follow-up.

Assessment of RR(RECIST) was practicable in only 30 of the 38 patients (78.9%) due to missing measurable target lesions according to RECIST 1.1 criteria (Table 4). Biochemical PR was found in 15 of the 30 patients, which was correctly correlated according to RR(RECIST) in six patients (40%). The rate of concordance between RR(RECIST) and BR was 45.5% (5/11 patients) for SD and only 25% for PD.

### Factors associated with overall survival

**Fig. 3:**
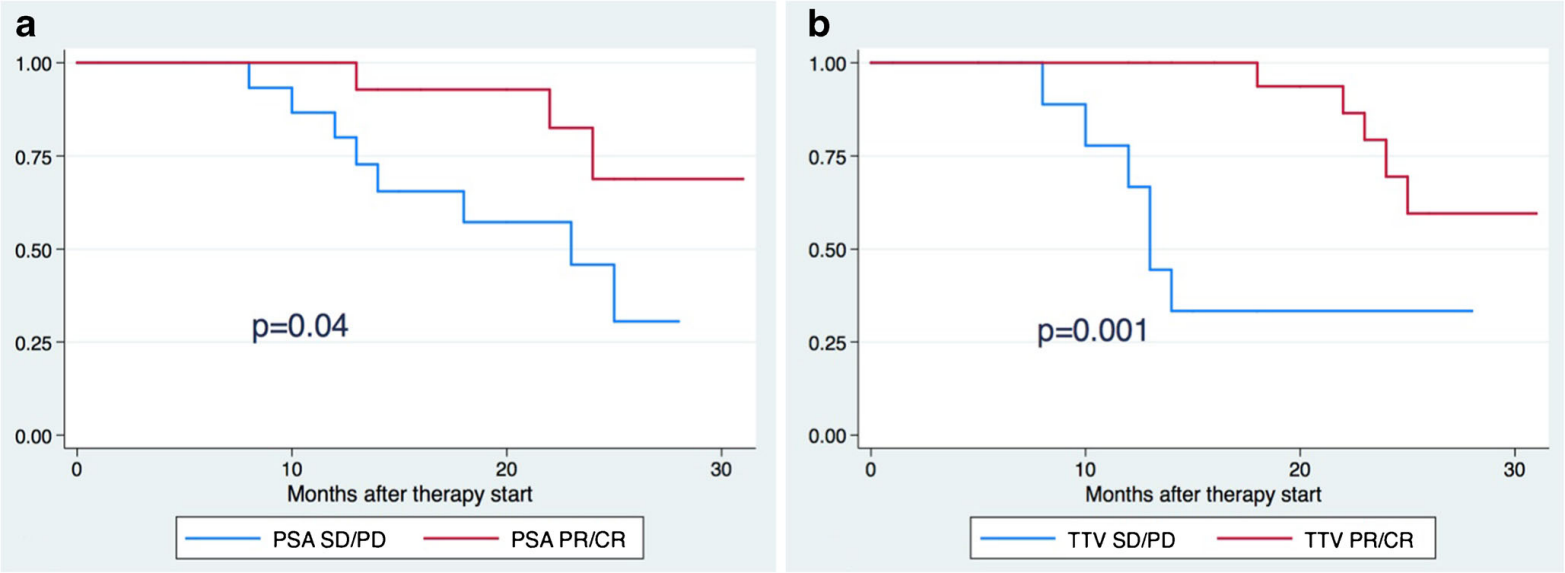
Kaplan-Meier analysis of overall survival in 38 patients with metastatic prostate cancer undergoing ^177^Lu-PSMA-617 radioligand therapy (RLT). **a** According to PSA response after RLT: *blue line* patients with biochemical stable disease (SD) or progressive disease (PD); *red line* patients with a biochemical partial response (PR) or complete response (CR). **b** According to total tumor volume (TTV) after RLT: *blue line* patients with stable disease (SD) or progressive disease (PD); *red line* patients with a partial response (PR) or complete response (CR)

After a median follow-up of 17 months (IQR 8.0, 24.2 months), 11 patients (28.9%) had died of their PC. In the univariable survival analysis, neither change in SUVmean nor the RECIST evaluation was associated with OS, whereas changes in TTV and PSA level were (HR 1.001, 95% CI 1–1.003, *p* = 0.04, and HR 1.004, 95% CI 1.001–1.008, *p* = 0.01, respectively). The Kaplan-Meier analysis also showed a significant difference in OS between patients with a PSA and/or TTV response of CR or PR and patients with SD or PD (*p* = 0.04 for PSA, *p* = 0.001 for TTV; Fig. 3). The pretreatment CRP value was also associated with OS (HR 1.07, 95% CI 1.009–1.14, *p* = 0.02), while the other pretreatment values (PSA level, hemoglobin, LDH, ALP) were not. Beyond that, none of the chosen pretreatment factors was associated with either TTV or PSA response in our investigated cohort.

## Discussion

Accurate assessment of therapy response is necessary for making optimal decisions regarding treatment allocation and schemes in patients with any type of disease, but specifically late stage cancers such as mCRPC. After having identified the potentially most effective and safe treatment, monitoring of response is necessary to allow changes in strategy early when the disease burden is relatively small. Unfortunately, accurate prediction of treatment response using the recommended monitoring tools including imaging modalities remains challenging [9]. PSMA imaging is recommended for assessing the PSMA expression of the tumor and its metastases is considered essential in determining the suitability of a patient for PSMA therapy (and also for commencement of further cycles), and is also valuable for, among other things, assessing tumor response rather relying on a simple serum blood marker. In addition, serum PSA has limitations for assessing therapeutic response, especially when patients are still receiving androgen-deprivation therapy, with regard to OS as the ultimate endpoint. This was also shown, for example, in the ALSYMPCA trial, which showed a survival benefit of ^223^Ra treatment in patients with castration-resistant prostate cancer [5]. Furthermore, PSMA imaging provides information about metastatic sites and spread, which opens a window for other targeted therapies, such as radiation therapy or even surgery for reducing tumor burden or preventing severe functional limitations, such as bone fractures.

The use of PSMA PET for primary staging and the detection of biochemical relapse has already been shown to lead to treatment alterations [13, 22]. PSMA imaging might therefore also enable new combined therapeutic approaches in the setting of castration resistance, which, of course, needs further evaluation. The results of the current study suggest that a hybrid imaging technique combining morphological and functional information, such as PSMA 11 PET/CT(MRI) may improve response assessment in mCRPC patients. This is in accordance with the findings of preclinical studies showing that PSMA uptake in the tumor is directly associated with the number of tumor cells and that decreased PSMA uptake after therapy is not due to treatment-induced changes but rather reliably reflects the number of living tumor cells [23]. Although several studies have shown adequate detection of treatment response for other radiopharmaceuticals [24, 25], the available data for PSMA 11 PET in mCRPC are still limited. The first study, to our knowledge, to investigate the use of PSMA PET for the assessment of response recently showed that PSMA PET performs better than conventional imaging in mCRPC patients undergoing systemic taxane-based chemotherapy [16]. A further study by Schmuck et al. introduced the parameter “PSMA-derived tumor volume” (PSMA-TV), which was obtained using an isocontour-based procedure from each lesion, and showed a correlation with PSA levels in an initial ten patients before and after therapy [26]. Our aims were to compare the predictive and monitoring accuracy of PSMA 11 PET with those of conventional imaging in patients after RLT using the BR as the standard of reference. We also assessed the association between RR including blood-based standard biomarkers and OS.

We used a simplified whole-body measurement of TTV which could be used as a routine clinical approach. Furthermore, the rationale for using this semiautomatic whole-body quantification based on a lower threshold of a standardized cubic liver VOI took into account the proposed recommendations for assessing PSMA 11 PET in prostate cancer [26]. Therefore, lesions with higher uptake than the liver background have to be assessed as suspicious for metastasis. Nevertheless, minor manual corrections are still needed for physiological uptake in the gut, liver, spleen, salivary glands and kidneys. The difference in liver background uptake before and after RLT was not significant (Table 2).

We found that PSMA PET parameters had a high accuracy in assessing response in mCRPC patients. To the best of our knowledge, this is the first study providing PSMA PET data on response to RLT and the first study in which PSMA TTV was used as an additional PET parameter. We also evaluated the correlation between RR and BR. Change in TTV showed a significant correlation with BR (*p* < 0.001), in contrast to change in SUVmean (*p* = 0.154) and the RECIST evaluation (*p* = 0.583). A previous study by Seitz et al. [16] assessed patients before and after docetaxel chemotherapy using the PSMA SUVmean. The rate of concordance between BR and RR(PET) was found to be higher than that between BR and RR(CT) in patients with castration-sensitive PC (86% vs. 50%, respectively) and in patients with castration-resistant PC (56% vs. 33%, respectively). Nevertheless, these results did not reach statistical significance. We could show comparable data to the study published before. Concordance rates for PSMA PET (TTV) and MRI(CT) for detecting PR were 83.3% and 40%, for detecting SD were 41.6% and 45%, and for detecting PD were 50% and 25%, respectively.

In eight patients response assessment was not possible with MRI or CT due to missing measurable lesions. Especially in metastatic prostate cancer, even the use of RECIST in combination with BS, as recommended by the Prostate Cancer Clinical Trials Working Group 3 [8], still does not overcome these known limitations. As BS might detect osteoblastic metastases earlier than changes on CT or MRI, BS might be detecting only a surrogate for osteoblastic activity caused by cancer cells, whereas PSMA-targeted imaging reveals the tumor cell itself. PSMA-targeted imaging might therefore be able to detect disease and therapy-related changes earlier, which is another argument for using hybrid imaging techniques such as PSMA 11 PET/MRI(CT) in mCRPC patients [27]. Interestingly and in contrast to data published before, changes in SUVmean seemed not to be superior to RECIST evaluation for response assessment, although neither of these rates reached a statistically significant association with PSA level as the standard of reference. This result suggests to a certain extent that repeated RLT reduces tumor burden but has only limited effect on the PSMA expression of the remaining tumor cells. Even the probable effect of decreased cell density due to RLT in these areas does not appear to have a significant impact on therapy response. Therefore, repeated RLT with six or even nine administrations still remains an option in these patients, as has already been shown by other groups [28].

PSMA TTV not only showed excellent concordance rates in response assessment, but its change was also associated with OS in the univariable survival analysis even in the small cohort of patients presented (HR 1.002, 95% CI 1–1.003, *p* = 0.03). The change in PSA levels also showed a significant association with OS (HR 1.004, 95% CI 1.001–1.008, *p* = 0.01). Such an association was not shown for the change in PSMA SUVmean and RR(RECIST). These findings were confirmed by the Kaplan-Meier analysis (Fig. 3), which showed a significant difference in OS between patients with TTV CR and PR and those with SD and PD. This was also true for BR but at a lower level of significance. Evaluating the relationship between PET parameters and objective clinical outcomes such as OS may have a major clinical impact in the future and might encourage further development of image assessment tools such as those used in this study.

We also evaluated pretreatment factors that have been found in previous studies to be associated with outcome in mCRPC patients (PSA, CRP, LDH and ALP levels, and hemoglobin) [17–19]. However, of all the known factors, only CRP level was significantly associated with OS in our cohort. The impact of RLT on progression-free survival and OS is not yet known, so that known predictors in the scope of other treatments might not apply for RLT, which could explain these controversial findings compared to those of other studies. Almost 50% of patients had a biochemical PR after RLT and about 30% had biochemical SD, which are BR rates that have not been found previously in this stage of the disease following other systemic therapies. This also explains our limited number of events (11 deaths) in the survival analyses. Nevertheless, this number represents almost one third of the cohort and was statistically significant.

The small number of patients and its retrospective design are limitations of this study. These factors also prevented a robust multivariable analysis, which is mandatory for the identification of independent predictors. This implies, that the preliminary associations with OS found have to be interpreted with caution. Furthermore, there are no known standardized criteria for response assessment using PSMA PET. The modified PERCIST 1.0 criteria [21] used in our study were initially developed for FDG PET. Another limitation is that RECIST evaluation was possible in only 30 of 38 patients (78.9%), because bone metastases are the most common manifestation of progressing mCRPC. For this reason the response in an unknown high number of patients may have been misclassified using RECIST, and BS was not carried out although recommended by the Prostate Cancer Clinical Trials Working Group 3 [8]. This can be attributed to the retrospective design of this study. PSMA PET has widely replaced regular bone scan in our clinic for screening patients for PSMA therapy. Therefore, the relationship between bone scans and PSMA therapy could not be analysed in this study because an insufficient number of scans was available.

Most of the patients (*n* = 32) were investigated using an integrated PET/MRI system. However, six patients were investigated with PSMA 11 PET/CT because of contraindications to MRI. Thus there are several concerns with regard to semiquantification. Most of the concerns are related to attenuation correction of bone lesions in PET/MRI systems using Dixon-based μ-maps, the generally lower SUV in PET/MRI systems, and follow-up examinations on different scanners [29]. To overcome some of these issues, we used a reference VOI for threshold-based quantification and all patients had their pretherapy and follow-up examinations on the same system, either PET/MRI or PET/CT. Another limitation is the use of serum PSA levels as the standard of reference for response assessment, as PSA levels have been shown to be not always reliable enough for monitoring disease activity in mCRPC patients. Nevertheless, in our study the changes in serum PSA levels and PSMA TTV were associated with OS in the univariable survival analysis, as well as the pretreatment CRP value, indicating a reliable correlation for response assessment in our cohort.

### Conclusion

This study suggests that PSMA PET-derived TTV is a promising tool for evaluating therapy response in mCRPC patients undergoing RLT and outperforms conventional cross-sectional imaging. It was significantly associated with BR. Univariable survival analysis demonstrated a potential association with OS. Further prospective studies are necessary to confirm these promising preliminary results.

## References

[1] Tannock IF, de Wit R, Berry WR, Horti J, Pluzanska A, Chi KN (2004). Docetaxel plus prednisone or mitoxantrone plus prednisone for advanced prostate cancer. New Engl J Med.

[2] Bahl A, Oudard S, Tombal B, Özgüroĝlu M, Hansen S, Kocak I (2013). Impact of cabazitaxel on 2-year survival and palliation of tumour-related pain in men with metastatic castration-resistant prostate cancer treated in the TROPIC trial. Ann Oncol.

[3] Ryan CJ, Smith MR, de Bono JS, Molina A, Logothetis CJ, de Souza P (2013). Abiraterone in metastatic prostate cancer without previous chemotherapy. New Engl J Med.

[4] Beer TM, Armstrong AJ, Rathkopf DE, Loriot Y, Sternberg CN, Higano CS (2014). Enzalutamide in metastatic prostate cancer before chemotherapy. New Engl J Med.

[5] Parker C, Nilsson S, Heinrich D, Helle SI, O’Sullivan JM, Fosså SD (2013). Alpha emitter Radium-223 and survival in metastatic prostate cancer. New Engl J Med.

[6] Fendler WP, Rahbar K, Herrmann K, Kratochwil C, Eiber M (2017). 177Lu-PSMA radioligand therapy for prostate cancer. J Nucl Med.

[7] Eisenhauer EA, Therasse P, Bogaerts J, Schwartz LH, Sargent D, Ford R (2009). New response evaluation criteria in solid tumours: revised RECIST guideline (version 1.1). Eur J Cancer.

[8] Scher HI, Morris MJ, Stadler WM, Higano C, Basch E, Fizazi K (2016). Trial design and objectives for castration-resistant prostate cancer: updated recommendations from the Prostate Cancer Clinical Trials Working Group 3. J Clin Oncol.

[9] Cook GJR, Azad G, Padhani AR (2016). Bone imaging in prostate cancer: the evolving roles of nuclear medicine and radiology. Clin Transl Imaging.

[10] Hartenbach M, Hartenbach S, Bechtloff W, Danz B, Kraft K, Klemenz B (2014). Combined PET/MRI improves diagnostic accuracy in patients with prostate cancer: a prospective diagnostic trial. Clin Cancer Res.

[11] Lordick F, Ott K, Krause B-J, Weber WA, Becker K, Stein HJ (2007). PET to assess early metabolic response and to guide treatment of adenocarcinoma of the oesophagogastric junction: the MUNICON phase II trial. Lancet Oncol.

[12] Cheson BD, Fisher RI, Barrington SF, Cavalli F, Schwartz LH, Zucca E (2014). Recommendations for initial evaluation, staging, and response assessment of Hodgkin and non-Hodgkin lymphoma: the Lugano classification. J Clin Oncol.

[13] Grubmüller B, Baltzer P, D’Andrea D, Korn S, Haug AR, Hacker M (2017). 68Ga-PSMA 11 ligand PET imaging in patients with biochemical recurrence after radical prostatectomy – diagnostic performance and impact on therapeutic decision-making. Eur J Nucl Med Mol Imaging.

[14] Maurer T, Gschwend JE, Rauscher I, Souvatzoglou M, Haller B, Weirich G (2016). Diagnostic efficacy of 68Gallium-PSMA positron emission tomography compared to conventional imaging for lymph node staging of 130 consecutive patients with intermediate to high risk prostate cancer. J Urol.

[15] Kaittanis C, Andreou C, Hieronymus H, Mao N, Foss CA, Eiber M (2018). Prostate-specific membrane antigen cleavage of vitamin B9 stimulates oncogenic signaling through metabotropic glutamate receptors. J Exp Med.

[16] Seitz AK, Rauscher I, Haller B, Krönke M, Luther S, Heck MM (2017). Preliminary results on response assessment using 68Ga-HBED-CC-PSMA PET/CT in patients with metastatic prostate cancer undergoing docetaxel chemotherapy. Eur J Nucl Med Mol Imaging.

[17] Zhao J, Sun G, Liao B, Zhang X, Armstrong CM, Yin X (2018). Novel nomograms for castration-resistant prostate cancer and survival outcome in patients with de novo bone metastatic prostate cancer. BJU Int.

[18] Liao S-G, Cheng H-H, Lei Y (2016). C-reactive protein is a prognostic marker for patients with castration-resistant prostate cancer. Oncol Res Treat.

[19] Mori K, Kimura T, Onuma H, Kimura S, Yamamoto T, Sasaki H (2017). Lactate dehydrogenase predicts combined progression-free survival after sequential therapy with abiraterone and enzalutamide for patients with castration-resistant prostate cancer. Prostate.

[20] Eiber M, Herrmann K, Calais J, Hadaschik B, Giesel FL, Hartenbach M (2018). Prostate cancer molecular imaging standardized evaluation (PROMISE): proposed miTNM classification for the interpretation of PSMA-ligand PET/CT. J Nucl Med.

[21] Wahl RL, Jacene H, Kasamon Y, Lodge MA (2009). From RECIST to PERCIST: evolving considerations for PET response criteria in solid tumors. J Nucl Med.

[22] Grubmüller B, Baltzer P, Hartenbach S, D’Andrea D, Helbich TH, Haug AR, et al. PSMA ligand PET/MRI for primary prostate cancer: staging performance and clinical impact. Clin Cancer Res. 2018. 10.1158/1078-0432.CCR-18-0768. 10.1158/1078-0432.CCR-18-0768 30139879

[23] Hillier SM, Kern AM, Maresca KP, Marquis JC, Eckelman WC, Joyal JL (2011). 123I-MIP-1072, a small-molecule inhibitor of prostate-specific membrane antigen, is effective at monitoring tumor response to taxane therapy. J Nucl Med.

[24] De Giorgi U, Caroli P, Burgio SL, Menna C, Conteduca V, Bianchi E (2014). Early outcome prediction on 18F-fluorocholine PET/CT in metastatic castration-resistant prostate cancer patients treated with abiraterone. Oncotarget Impact J.

[25] Ceci F, Castellucci P, Graziani T, Schiavina R, Renzi R, Borghesi M (2015). 11C-choline PET/CT in castration-resistant prostate cancer patients treated with docetaxel. Eur J Nucl Med Mol Imaging.

[26] Schmuck S, von Klot CA, Henkenberens C, Sohns JM, Christiansen H, Wester HJ (2017). Initial experience with volumetric 68Ga-PSMA I&T PET/CT for assessment of whole-body tumor burden as a quantitative imaging biomarker in patients with prostate cancer. J Nucl Med.

[27] Pyka T, Okamoto S, Dahlbender M, Tauber R, Retz M, Heck M (2016). Comparison of bone scintigraphy and 68Ga-PSMA PET for skeletal staging in prostate cancer. Eur J Nucl Med Mol Imaging.

[28] Rathke H, Giesel FL, Flechsig P, Kopka K, Mier W, Hohenfellner M (2018). Repeated 177Lu-labeled PSMA-617 radioligand therapy using treatment activities of up to 9.3 GBq. J Nucl Med.

[29] Aide N, Lasnon C, Veit-Haibach P, Sera T, Sattler B, Boellaard R (2017). EANM/EARL harmonization strategies in PET quantification: from daily practice to multicentre oncological studies. Eur J Nucl Med Mol Imaging.

